# Supplementary material for: Ballistic transport spectroscopy of spin-orbit-coupled bands in monolayer graphene on WSe2
Source: Nat Commun. 2023 Sep 30;14:6124. doi: 10.1038/s41467-023-41826-1 (PMC10542375; doi:10.1038/s41467-023-41826-1)
Supplement: Supplementary file 1 — Supplementary Information [file 41467_2023_41826_MOESM1_ESM.pdf]

# Ballistic transport spectroscopy of spin-orbit-coupled bands in monolayer graphene on $WSe_2$

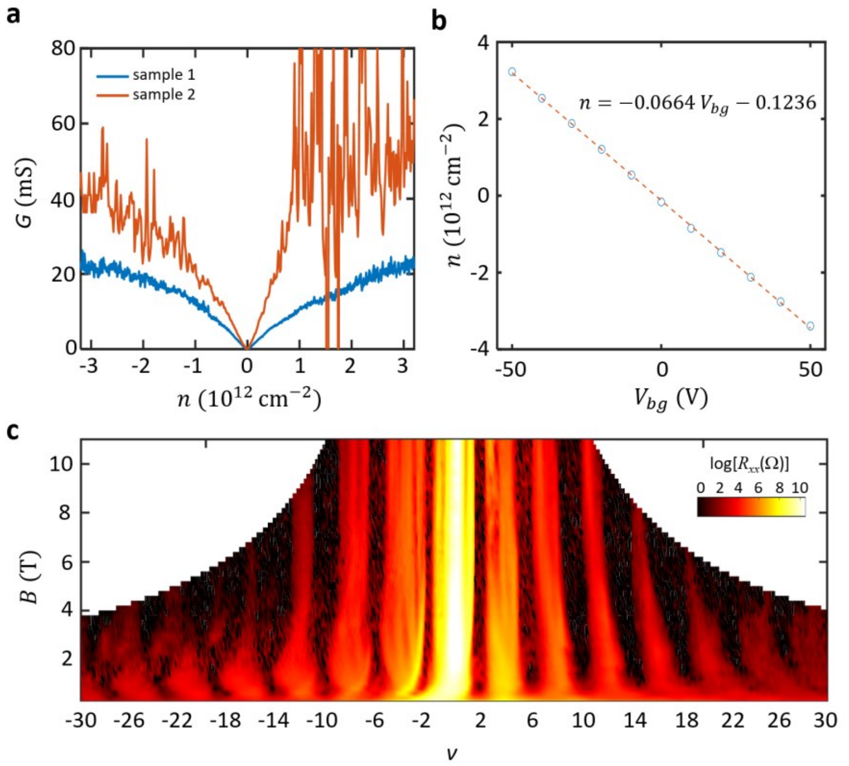


**Supplementary Figure 1. Additional data on sample characteristics. a** The four-terminal conductance $G$ as a function of the carrier density $n$. For sample 2, the conductance shows abrupt saturation and large fluctuations at around $n > 1 \times 10^{12}\ cm^{-2}$ while for sample 1, no obvious change is found in the density range we measured. The abrupt change of the conductance and large fluctuations come from the $WSe_2$ flake being conducting that pinches off the transport through graphene by sinking its charge carriers as found in various studies[1-5]. **b** The Hall density as a function of the gate voltage for device 1, where the linear fitting gives the areal capacitance divided by electric charge, $C_{bg}/e = 6.6\times10^{10}$ cm$^{-2}$ V$^{-1}$ with $C_{bg} = 1.056 \times 10^{-4}$ Fm$^{-2}$. From this value, the charge density can be obtained, $n = C_{bg}/e \times \Delta V_{bg}$ with $\Delta V_{bg} \equiv V_{bg} - V_{CNP}$ ($V_{CNP}$: $V_{bg}$ at the charge neutrality point). The Hall density at each gate is obtained from the slope of each $R_{xy} - B$ curve. **c** The color map of log($R_{xx}$) as a function of Landau level filling factor $\nu$ and magnetic field $B$, calculated by the capacitance value extracted from classical Hall effect in **a**. $R_{xx}$ minima occur at $\nu = \pm2,\ \pm6,\ \pm10,\ \pm14$, matching with the expected position of quantum Hall plateau for monolayer graphene.

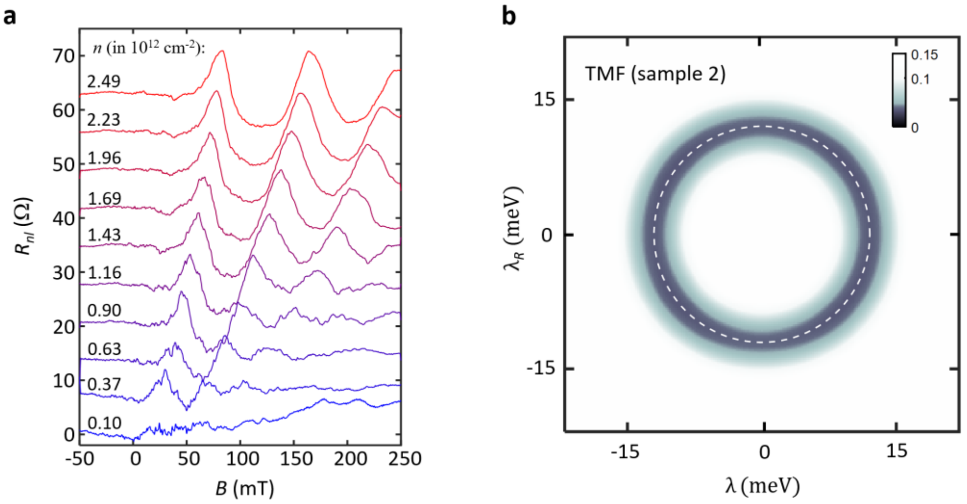


**Supplementary Figure 2. Additional analysis on TMF spectra. a** 1D cuts of the data from sample 1 on the electron side shown in Fig. 2a of the main text. **b** The color-scale map of the average difference $\langle \delta B^2 \rangle \equiv \sum[(\Delta B_+/B_0)^2 + (\Delta B_-/B_0)^2]/N$ as a function of $\lambda$ and $\lambda_R$ from sample 2 (see the caption of Fig. 3a for details).

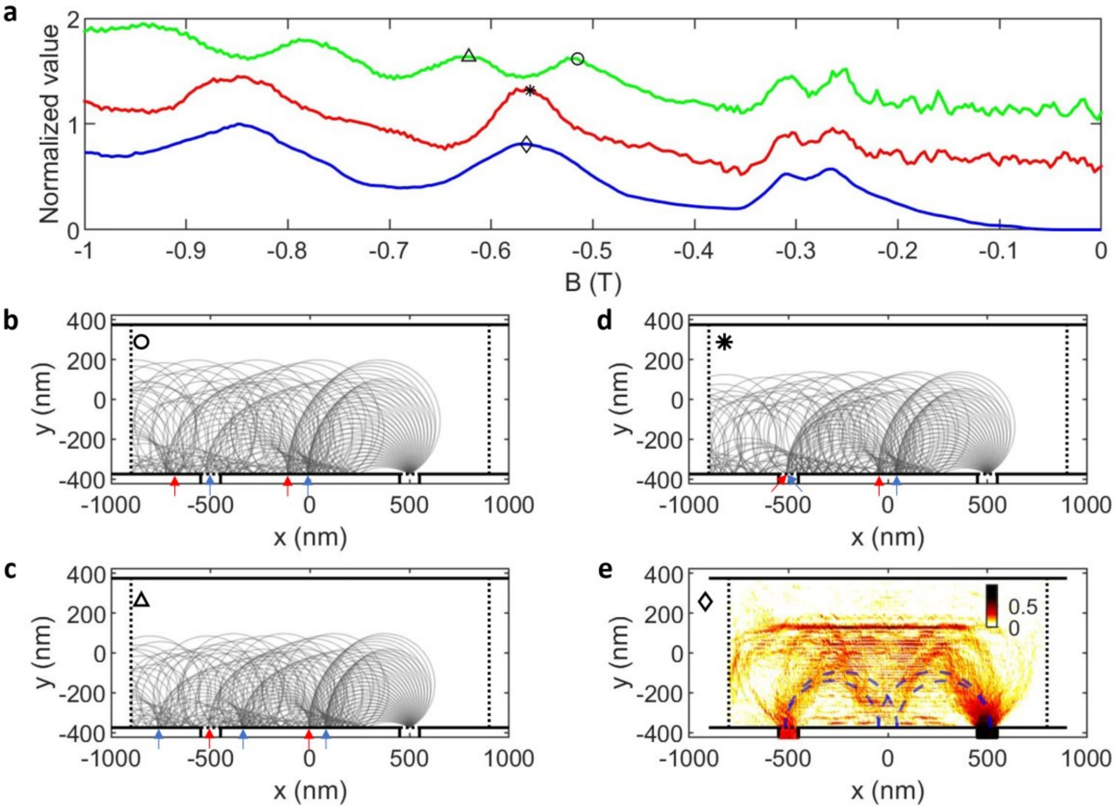


**Supplementary Figure 3. Inter-band transition simulation for the second focusing peak. a** Top green and middle red curves: normalized TMF conductance calculated by semi-classical ray tracing without or with inter-band transition, respectively. Bottom blue curve: normalized conductance from quantum transport simulation. As expected, the second focusing peak shows splitting when the inter-band transition is switched off (top green curve). Moreover, the positions of the focusing peaks calculated with inter-band transition (middle red curve) match well with those from Kwant simulation (bottom blue curve), indicating that the observed absence of the splitting in the second focusing peak (Figs. 2a-c) is from the inter-band transition. **b-d** The calculated carrier trajectories at the focusing peaks marked by circle, triangle, and star in **a**, respectively. Red and blue arrows indicate the positions of the scattering at the edge for carriers at $S_\pm$ bands, respectively. **e** Local current density distribution at the focusing peak marked by diamond from quantum transport simulation. Dashed lines illustrate the carrier trajectories.

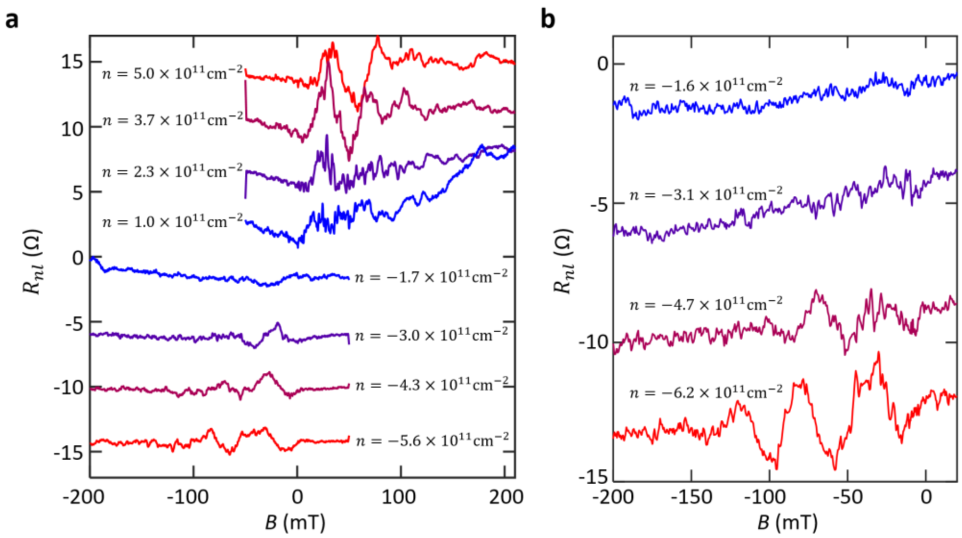


**Supplementary Figure 4. TMF spectra near zero density.** 1D cuts of the data shown in Figs. 2a,b for sample 1 (**a**) and 2 (**b**). No TMF signals are observed below around $2{\sim}3 \times 10^{11}\ cm^{-2}$ which matches roughly with the density below which negative non-local resistance disappears (the inset of Fig. 1e) and large non-local signal appears (Fig. 4b). It indicates the crossover between the diffusive spin-Hall and ballistic TMF effects.

## Supplementary Note 1. Quantum transport simulation

Current density maps (the inset of Fig. 1c and Supplementary Fig. 3e) and conductance spectra (Fig. 1d, right) were simulated using Kwant[6], an open-source python package for quantum transport simulations based on tight-binding models. To model transport in graphene on TMDCs, we adopt the effective tight-binding Hamiltonian[7]

$$H = H_0 + H_1 + H_R + H_{vZ} + H_{PIA}, \quad \text{(S1)}$$

where the first two terms are spin-independent and the rest describe spin-orbit couplings of different origins. The first term, $H_0 = \sum_{\langle i,j \rangle,\sigma} t c_{i\sigma}^{\dagger} c_{j\sigma}$, is composed of nearest-neighbor kinetic hopping of strength $t$, and is typically used to describe a spinless graphene lattice. Here, $i, j$ are lattice site indices, $\sigma$ is the spin index, $c_{i\sigma}^{\dagger}$ ($c_{j\sigma}$) is the creation (annihilation) operator that creates (annihilates) an electron of spin $\sigma$ at site $i$, and $\Sigma_{\langle i,j \rangle}$ sums over site indices that are nearest to each other. Except $H_0$, all the rest of the terms in Eq. (S1) arise from the effect of the neighboring TMDC lattice. First, the symmetry between the sublattices A and B of the graphene lattice is broken, giving rise to an effective energy difference experienced by electrons on atoms of the two sublattices, which can be described by

$$H_1 = \sum_{i,\sigma} \xi_{o_i} \Delta c_{i\sigma}^{\dagger} c_{i\sigma} ,$$

where $o_i$ is the sublattice index of site $i$, $\xi_{oi} = +1\ (-1)$ when $o_i = A$ ( $o_i = B$ ), and $\Delta$ characterizes the strength of such a staggered potential energy. The rest of the three terms in Eq. (2) of the main text include the Rashba spin-orbit coupling,

$$H_R = \frac{2i}{3} \sum_{\langle i,j \rangle} \sum_{\sigma,\sigma'} c_{i\sigma}^{\dagger} c_{j\sigma'} \left[ \lambda_R \left( \boldsymbol{s} \times \boldsymbol{d}_{ij} \right)_z \right]_{\sigma,\sigma'} ,$$

where $\lambda_R$ the Rashba coupling strength, $\boldsymbol{s} = (\boldsymbol{s}_x, \boldsymbol{s}_y, \boldsymbol{s}_z)$ is a vector of Pauli matrices acting on spin, and $\boldsymbol{d}_{ij}$ is a unit vector pointing from site $j$ to $i$, the valley-Zeeman term,

$$H_{vZ} = \frac{i}{3\sqrt{3}} \sum_{\langle\langle i,j \rangle\rangle} \sum_{\sigma,\sigma'} c_{i\sigma}^{\dagger} c_{j\sigma} \left[ \lambda_I^{o_i} \nu_{ij} \boldsymbol{s}_z \right]_{\sigma,\sigma'} ,$$

where $\Sigma_{\langle\langle i,j \rangle\rangle}$ sums over site indices $i, j$ that are second nearest to each other, the sign factor $\nu_{ij} = +1\ (-1)$ when the resulting hopping path is counterclockwise (clockwise), and $\lambda_I^{o_i}$ is the sublattice-resolved valley-Zeeman coupling strength, and finally the pseudospin-inversion-asymmetry term $H_{PIA}$ that does not influence the band structure at $K$ and $K'$. Neglecting $H_{PIA}$ and setting $\lambda_I^A = -\lambda_I^B = \lambda$ for both sublattices $o_i = A, B$, the eigenenergy of tight-binding model Hamiltonian Eq. (3) is given by[8]

$$E_{\mu,\nu}(k) = \mu \sqrt{(\Delta^2 + \lambda^2 + 2\lambda_R^2 + \hbar^2 v_F^2 k^2) + 2\nu \sqrt{(\lambda_R^2 - \lambda\Delta)^2 + (\lambda^2 + \lambda_R^2)\hbar^2 v_F^2 k^2}}$$

with $\mu, \nu = \pm 1$, whose low-$k$ expansion simplifies to the eigenenergy of Eq. (1) of the main text, which is adopted in several previous studies[1,8-10]. Note that to account for micron-sized graphene systems, we have adopted the scaled tight-binding model[11] which is compatible with the spin-orbit coupling terms as remarked in the recent study[12]. All quantum transport simulations presented here are based on the scaling factor $s_F = 8$.

To simulate our TMF experiment done on the multi-terminal graphene Hall bar (Fig. 1b of the main text), considering the geometry as close to the real device as possible while keeping the computation affordable, we calculate the conductance $G$ between the injector and collector (those reported in Figs. 1d and 3f), i.e., $G = T(e^2/h)$ where $T$ is the corresponding transmission function (from the injector lead to the collector lead), based on the Landauer formula[13]. On the other hand, the experimentally measured TMF signal is based on the so-called four-point resistance (such as Fig. 2 of the main text), which can also be simulated using the Büttiker formula[13]. Computationally, however, the Büttiker formula requires computing $N(N-1)$ transmission functions between any pair of two different leads for an $N$-terminal system.

In our case (see Fig. 1b of the main text), there are $10 \times 9 = 90$ transmission functions to compute, which is beyond our computation limit, even if we could afford to model the full size of our experimental device. In fact, instead of modeling the full device of our experiment, we used an effective three-terminal device of smaller area and shorter probe spacing ($L = 1.0$ μm) for our transport simulations, in order to lower the computation burden. Nevertheless, our conductance calculations (for the effective three-terminal Hall bar) reveal consistent behaviors of the TMF peaks, compared to those from our four-point resistance measurement, including the splitting of the first peaks.

## Supplementary Note 2. Semi-classical ray tracing

In order to check if the absence of the splitting in the second focusing peak (Figs. 1d and 2) is due to the inter-band scattering at the edge, we have employed the semi-classical ray tracing by solving the following equation of motion[14]:

$$\begin{cases} \boldsymbol{v} = \dfrac{1}{\hbar}\nabla_{\boldsymbol{k}} E(\boldsymbol{k}) \\ \dfrac{d\boldsymbol{k}}{dt} = -\dfrac{e}{\hbar}\boldsymbol{v} \times \boldsymbol{B} \end{cases},$$

where one can manually add or remove scattering conditions like the inter-band transition at the edge. Supplementary Fig. 3 shows the results that clearly prove that the absence of the splitting in the second focusing peak is from the scattering between the two bands $S_+$ and $S_-$ at the sample edge.

**Supplementary Note 3. Discussions on TMF and SdH oscillations**

As discussed briefly in the main text, in TMF, the electrons make only half of the cyclotron motion while in SdH oscillations, it needs to make a full circle without losing its phase coherence. This can give rise to several differences in the two phenomena. First, the TMF is more sensitive to the electron scattering than the SdH oscillations as TMF is from the ballistic motion of electrons that can be destroyed by elastic scattering. Thus, in our sample, we could see the splitting in TMF focusing peak only on the hole side where we find higher sample quality while in SdH oscillations we found no obvious differences in both hole and electron side. Second, as electrons need to make a full cyclotron motion, SdH oscillations probe the total Fermi surface area only, whereas the TMF probes the partial trajectory along the Fermi surface. This can make a difference when the band structure is shifted in one momentum direction while keeping its area. This might be the reason why the studies on 2DEG with SOC including ours showed different splitting in TMF and SdH oscillations. Lastly, the SdH oscillations generally occur at larger magnetic fields than the TMF. This may result in a stronger effect of the Zeeman energy that couples to out-of-plane spin components. It could thus affect the spin-valley Zeeman and Rashba terms in Eq. (1) differently, that may lead to different band splitting in SdH oscillations compared with TMF. Nonetheless, the microscopic process that governs TMF and SdH oscillations is different and our study, together with other studies on 2DEG with SOC[15,16], shows that it might be important to consider their differences when analysing the relevant experimental results.

## Supplementary References


1 Wang, Z. *et al.* Strong interface-induced spin–orbit interaction in graphene on $WS_2$. *Nat. Commun.* **6**, 8339 (2015).

2 Wang, Z. *et al.* Origin and Magnitude of `Designer' Spin-Orbit Interaction in Graphene on Semiconducting Transition Metal Dichalcogenides. *Phys. Rev. X* **6**, 041020 (2016).

3 Avsar, A. *et al.* Spin-orbit proximity effect in graphene. *Nat. Commun.* **5**, 4875 (2014).

4 Yang, B. *et al.* Strong electron-hole symmetric Rashba spin-orbit coupling in graphene/monolayer transition metal dichalcogenide heterostructures. *Phys. Rev. B* **96**, 041409 (2017).

5 Zihlmann, S. *et al.* Large spin relaxation anisotropy and valley-Zeeman spin-orbit coupling in $WSe_2$/graphene/*h*-BN heterostructures. *Phys. Rev. B* **97**, 075434 (2018).

6 Groth, C. W., Wimmer, M., Akhmerov, A. R. & Waintal, X. Kwant: a software package for quantum transport. *New. J. Phys.* **16**, 063065 (2014).

7 Gmitra, M., Kochan, D., Högl, P. & Fabian, J. Trivial and inverted Dirac bands and the emergence of quantum spin Hall states in graphene on transition-metal dichalcogenides. *Phys. Rev. B* **93**, 155104 (2016).

8 Zubair, M., Vasilopoulos, P. & Tahir, M. Influence of interface induced valley-Zeeman and spin-orbit couplings on transport in heterostructures of graphene on $WSe_2$. *Phys. Rev. B* **101**, 165436 (2020).

9 Gmitra, M. & Fabian, J. Graphene on transition-metal dichalcogenides: A platform for proximity spin-orbit physics and optospintronics. *Phys. Rev. B* **92**, 155403 (2015).

10 Tiwari, P. *et al.* Experimental observation of spin−split energy dispersion in high-mobility single-layer graphene/$WSe_2$ heterostructures. *npj 2D Mater. Appl.* **6**, 68 (2022).

11 Liu, M.-H. *et al.* Scalable Tight-Binding Model for Graphene. *Phys. Rev. Lett.* **114**, 036601 (2015).

12 Zhumagulov, Y., Frank, T. & Fabian, J. Edge states in proximitized graphene ribbons and flakes in a perpendicular magnetic field: Emergence of lone pseudohelical pairs and pure spin-current states. *Phys. Rev. B* **105**, 205134 (2022).

13 Datta, S. *Electronic Transport in Mesoscopic Systems* (Cambridge University Press, 1995).

14 Ashcroft, N. W. & Mermin, N. D. *Solid state physics*. (Holt, 1976).

15 Lo, S.-T. *et al.* Controlled spatial separation of spins and coherent dynamics in spin-orbit-coupled nanostructures. *Nat. Commun.* **8**, 15997 (2017).

16 Rendell, M. J. *et al.* Gate voltage dependent Rashba spin splitting in hole transverse magnetic focusing. *Phys. Rev. B* **105**, 245305 (2022).
